# Clinical efficacy and safety of adjuvant EGFR‐TKIs for resected stage IB lung adenocarcinoma: A real‐world study based on propensity score matching

**DOI:** 10.1002/cam4.6443

**Published:** 2023-08-09

**Authors:** Leilei Shen, Juntang Guo, Weidong Zhang, Lianbin Zhang, Xi Liu, Tao Wang, Tao Zhang, Chaoyang Liang, Yang Liu

**Affiliations:** ^1^ Postgraduate School Medical School of Chinese PLA Beijing People's Republic of China; ^2^ Department of Thoracic Surgery Chinese PLA General Hospital Beijing People's Republic of China; ^3^ Department of Thoracic Surgery Hainan Hospital of Chinese PLA General Hospital Sanya People's Republic of China; ^4^ Department of Thoracic Surgery The First Medical Center of Chinese PLA General Hospital Beijing People's Republic of China

**Keywords:** adenocarcinoma, adjuvant therapy, disease‐free survivals, EGFR‐TKIs, stage IB

## Abstract

**Background:**

Adjuvant therapy for stage IB non‐small cell lung cancer remains debatable. In this real‐world study, we evaluate the efficacy and safety of adjuvant epidermal growth factor receptor tyrosine kinase inhibitors (EGFR‐TKIs) for resected stage IB lung adenocarcinoma.

**Methods:**

This real‐world study recruited 249 patients diagnosed with stage IB disease after surgical resection between January 2013 and September 2021. Sixty‐six (26.5%) patients received adjuvant targeted therapy (TKIs group), and 183 (73.5%) were enrolled in the clinical observation (CO) group. Propensity scores were matched to minimize the observed confounder effects between the two groups, and 59 patient pairs were matched. The primary endpoint was disease‐free survival (DFS).

**Results:**

In the TKI group, 38 (64.4%) patients chose to receive icotinib, 27.1% (16/59) received gefitinib, and 5 patients (8.5%) chose osimertinib. The median follow‐up time was 30.8 months (range: 7–107 months). Two (3.4%) patients in the TKI group and 10 (16.9%) in the CO group experienced disease relapse. The 3‐year DFS rates were 98.3% in the TKI group and 83.0% in the CO group (HR: 0.10; 95% CI: 0.01–0.78; *p* = 0.008). DFS differences were found in the entire cohort (*p* = 0.005) and the matched cohort (*p* = 0.024) between the two groups. Multivariate analysis showed that adjuvant EGFR‐TKIs was an independent factor for DFS (HR: 0.211; 95% CI: 0.045–0.979; *p* = 0.047), along with poor cell differentiation (HR: 5.256; 95% CI: 1.648–16.769; *p* = 0.005), and spread through air spaces (HR: 5.612; 95% CI: 1.137–27.700; *p* = 0.034). None of the patients discontinued EGFR‐TKIs owing to the low occurrence rate of treatment‐related serious adverse events.

**Conclusion:**

Adjuvant EGFR‐TKIs could significantly improve DFS among patients with stage IB lung adenocarcinoma compared with CO, with a safe and tolerable profile.

## 1 INTRODUCTION

Lung cancer is the leading cause of cancer‐related mortality, with adenocarcinoma accounting for 40%–55% of all lung cancers, manifesting as the most frequent subtype worldwide.^1^ Lobectomy with systemic nodal dissection remains the fundamental approach for patients with early stage non‐small cell lung cancer (NSCLC).^2^ However, only 16% of patients are diagnosed with stage I disease after radical resection.^3^ Among them, the 5‐year overall survival (OS) rate of stage IB is 68%, implying that approximately 30% of patients inevitably experience recurrence, metastasis, and death.^4^ Undetectable minimal residual disease (MRD) may contribute to relapse, and elimination of MRD with adjuvant therapy is greatly necessary.^5^ Previous studies have failed to show a significant survival benefit from platinum‐based adjuvant chemotherapy for stage IB NSCLC.^6^, ^7^, ^8^, ^9^, ^10^, ^11^, ^12^ Present guidelines, including those of the European Society for Medical Oncology (ESMO) and the American Society of Clinical Oncology, do not recommend adjuvant therapy for completely resected IB NSCLC.^13^, ^14^ Only the National Comprehensive Cancer Network (NCCN) guidelines recommend adjuvant chemotherapy or epidermal growth factor receptor tyrosine kinase inhibitors (EGFR‐TKIs) for high‐risk stage IB patients.^15^ Adverse effects and inconveniences limit the use of chemotherapy. Targeted therapy, particularly EGFR‐TKIs, has shown greater response rates and longer OS, compared with chemotherapy, in advanced NSCLC in the last decade,^16^, ^17^, ^18^ and it was recommended as the first‐line treatment for EGFR mutation‐positive advanced patients.^13^, ^14^, ^15^ Based on these positive outcomes, the ADAURA trial was the first study to investigate the efficacy and safety of third‐generation EGFR‐TKIs as an adjuvant option for resected stage IB patients, which suggested that adjuvant osimertinib may reduce the risk of relapse or death by 61% in subgroup analysis.^19^ Similarly, the CORIN study evaluated the use of icotinib in stage IB NSCLC patients and revealed that icotinib significantly improved the 3‐year disease‐free survival (DFS) rate, compared with the observation group.^20^ However, the ADAURA and CORIN studies used the 7th, instead of the latest 8th edition of the American Joint Committee on Cancer and Union International Contre le Cancer (AJCC/UICC) staging system for lung cancer.^4^, ^21^

Thus, we devised and conducted this real‐world study to evaluate the efficacy and safety of adjuvant EGFR‐TKIs for completely resected stage IB lung adenocarcinoma.

## 2 PATIENTS AND METHODS

### 2.1 Study population

**FIGURE 1:**
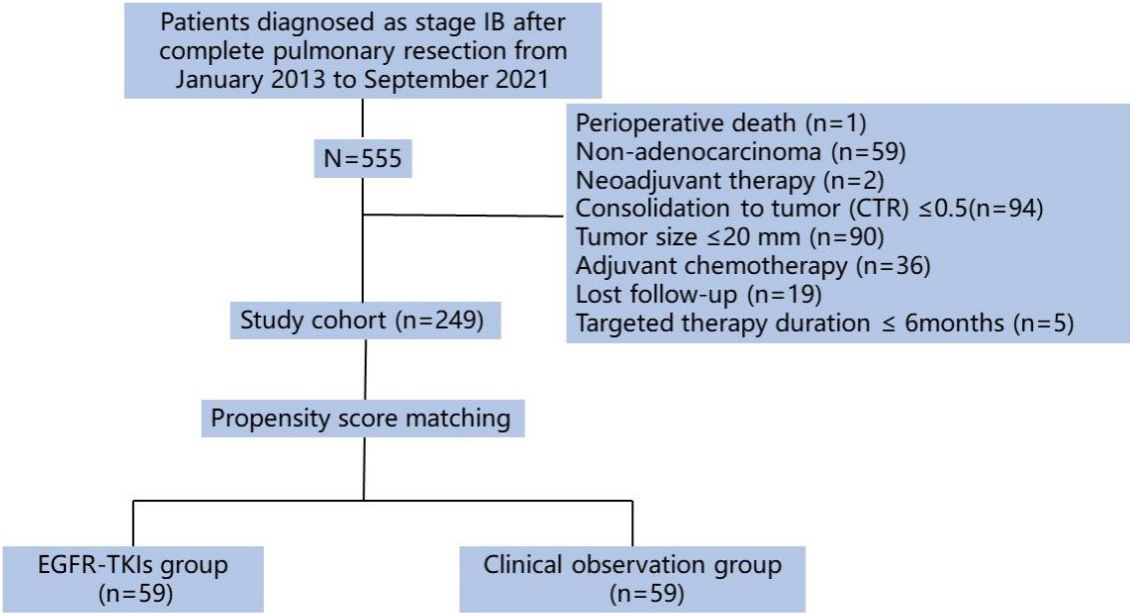
Study enrollment flow diagram. EGFR‐TKIs, epidermal growth factor receptor tyrosine kinase inhibitors.

We reviewed the patient records at our thoracic center between January 2013 and September 2021. The inclusion criteria were as follows: patients who underwent complete pulmonary resection (lobectomy or sublobectomy) with or without lymph node dissection; life expectancy of at least 1 year; confirmed consolidation tumor ratio (CTR) >0.5 or pure solid nodule in chest computed tomography (CT); adenocarcinoma tumor pathology,^22^ pathological stage of T2aN0M0 according to the 8th edition stage classification,^4^ and EGFR‐TKI administration longer than 1 year. The following patients were excluded: (1) those with metastatic lung tumors or distant metastasis; (2) those who received neoadjuvant therapy or adjuvant chemotherapy before EGFR‐TKIs; (3) those who were unable to comply with the protocol; (4) those who died within 30 days of surgery; and (5) those who were lost to follow‐up. Finally, 249 patients were enrolled in this study (Figure 1), and their clinicopathological characteristics and survival outcomes were collected from the hospital's electronic medical record system. Twenty percent of the mucous, micropapillary, and solid components were recorded as positive in the respective subtypes of adenocarcinoma and were considered to have low differentiation.

### 2.2 Procedures

Adjuvant treatment is recommended for stage IB patients, especially those with one or more of the following high‐risk factors: poor cell differentiation, micropapillary component, solid component, visceral pleural invasion (VPI), sublobectomy, unknown lymph node status (pNx), lymphovascular invasion (LVI), and spread through air spaces (STAS). They chose to receive EGFR‐TKIs or observation according to their will and EGFR mutation status. Patients who underwent targeted treatment showed EGFR‐activating mutations in exons 19 or 21 (either alone or in combination with other mutations) in genetic tests on tumor tissue. They were recommended to initiate with the drug within 6 weeks after surgery and to take medication for more than 1 year. Three options were offered: 80 mg osimertinib once daily, 250 mg gefitinib once daily, or 125 mg icotinib thrice daily. Adjuvant‐targeted therapy can be discontinued until disease relapse or when unacceptable adverse effects occur. The study was performed in accordance with the guidelines for Good Clinical Practice and the principles of the Declaration of Helsinki, and was approved by the Ethics Committee of the Chinese People's Liberation Army General Hospital (S2021‐701‐01). All patients signed an informed consent form.

### 2.3 Follow‐up

Routine examinations after surgery were requested every 3 months for the first 2 years, and every 6 months thereafter for 5 years. After 5 years, the patients were assessed annually. The examinations included blood tumor marker testing, chest CT, ultrasound of the neck and abdomen, and head magnetic resonance imaging. Bone scans were performed for patients with bone pain. Safety information, including clinical and laboratory adverse events, was also recorded for the EGFR‐TKI patients. Follow‐up information was collected through official contact with patients via telephone or from hospital outpatient clinic records. The last follow‐up was performed in August 2022. The primary endpoint was DFS, which was defined as the time from the surgery date to the first event (recurrence, metastasis, or NSCLC‐related death) or the last follow‐up. DFS was calculated in months.

### 2.4 Propensity score matching (PSM)

We conducted PSM to minimize the effects of observed confounders due to the imbalance of baseline characteristics between the TKI and clinical observation (CO) groups.^23^ Patients were matched based on the following covariates: age, sex, smoking history, pathological tumor diameter, CTR, poor cell differentiation, micropapillary component, solid component, VPI, LVI, STAS, surgical procedure (sublobectomy/lobectomy), and pNx. Patients who did or did not receive EGFR‐TKIs were matched 1:1 for propensity scores without replacement using a greedy nearest neighbor matching algorithm with a caliper distance of 0.02.

### 2.5 Statistical analysis

All statistical analyses were performed using SPSS version 22 (IBM Corporation). Continuous variables are expressed as means with standard deviation, as well as medians with a range of values. We used the Mann–Whitney *U* test to determine significant differences in continuous variables between the two groups. Every group with categorical variables was summarized using the frequency and percentage of the considered population, and statistical comparisons between the two groups were performed using the chi‐squared test. DFS curves were estimated using the Kaplan–Meier method, and the log‐rank test was used to compare differences. The Cox proportional hazards model was applied in univariate and multivariate analyses to determine its influence on patient risk of recurrence. A two‐sided *p* < 0.05 was considered statistically significant.

## 3 RESULTS

### 3.1 Clinical characteristics before and after PSM

Among the 555 patients diagnosed with stage IB disease after surgical resection between January 2013 and September 2021 at our thoracic center, 249 were recruited for this study (Table 1). Sixty‐six (26.5%) and 183 (73.5%) patients were enrolled in the TKI and CO groups, respectively. Differences were observed in sex (*p* = 0.002), smoking history (*p* = 0.003), carcinoembryonic abnormality (*p* = 0.008), solid nodule (*p* = 0.006), EGFR mutation (*p* < 0.001), and cell differentiation (*p* = 0.018) before PSM. The two groups were comparable after PSM.

**TABLE 1:** Patient clinical characteristics before and after PSM.

Characteristic	Entire cohort		*p*	PSM cohort		*p*
	TKI group	CO group		TKI group	CO group	
No.	66	183		59	59	
Sex			0.002			0.701
Male	22 (33.3)	101 (55.2)		22 (37.3)	20 (33.9)	
Female	44 (66.7)	82 (44.8)		37 (62.7)	39 (66.1)	
Age, years	61.03 ± 8.07	61.13 ± 8.97	0.936	60.51 ± 8.35	61.80 ± 8.70	0.414
<65	43 (65.2)	114 (62.3)	0.680	40 (67.8)	36 (61.0)	0.442
≥65	23 (34.8)	69 (37.7)	0.680	19 (32.2)	23 (39.0)	0.442
Smoking history			0.003			0.782
Yes	8 (12.1)	56 (30.6)		8 (13.6)	7 (11.9)	
No	58 (87.9)	127 (69.4)		51 (86.4)	52 (88.1)	
Family history of lung cancer	12 (18.2)	29 (15.8)	0.661	11 (18.6)	10 (16.9)	0.810
CEA abnormality	14 (21.2)	16 (8.7)	0.008	8 (13.6)	9 (15.3)	0.793
PET‐CT	4.6 (2.05, 8.75)	3.8 (2.1, 8.9)	0.655	3.9 (2.5, 6.7)	3.9 (3.0, 4.8)	0.569
Tumor location			0.061			0.284
RUL	14 (21.2)	45 (24.6)		11 (18.6)	14 (23.7)	
RML	13 (19.7)	14 (7.7)		11 (18.6)	6 (10.2)	
RLL	9 (13.6)	36 (19.7)		9 (15.3)	6 (10.2)	
LUL	19 (28.8)	48 (26.2)		18 (30.5)	20 (33.9)	
LLL	11 (16.7)	40 (21.9)		10 (16.9)	13 (22.0)	
CTR	0.99 (0.73, 1)	1 (0.83, 1)	0.056	1 (1, 1)	1 (0.82, 1)	0.500
Solid nodule	34 (51.5)	59 (32.2)	0.006	31 (52.5)	25 (42.4)	0.269
Surgical procedure			0.644			0.782
Sublobectomy	9 (13.6)	21 (11.5)		8 (13.6)	7 (11.9)	
Lobectomy	57 (86.4)	162 (88.5)		51 (86.4)	52 (88.1)	
LND						
Stations	5 (5, 6)	5 (5, 6)	0.487	5 (5, 6)	6 (4, 7)	0.651
Numbers	10.08 ± 7.17	11.02 ± 6.30	0.315	9.90 ± 7.29	10.09 ± 5.60	0.876
pNx	13 (19.7)	40 (21.9)	0.713	11 (18.6)	15 (25.4)	0.374
EGFR mutation	66 (100)	150 (82.0)	<0.001	59 (100)	55 (93.2)	0.119
Tumor diameter(mm)	27.24 ± 8.07	28.19 ± 6.63	0.397	26.81 ± 8.26	27.64 ± 6.48	0.545
>3 cm	29 (43.9)	94 (51.4)	0.301	25 (42.4)	30 (50.8)	0.356
≤3 cm	37 (56.1)	89 (48.6)	0.301	34 (57.6)	29 (49.2)	0.356
Mucous component	6 (9.1)	20 (10.9)	0.675	4 (6.8)	3 (5.1)	0.697
Micropapillary component	14 (21.2)	41 (22.4)	0.841	11 (18.6)	12 (20.3)	0.816
Solid component	6 (9.1)	32 (17.5)	0.104	5 (8.5)	4 (6.8)	0.729
VPI	49 (74.2)	144 (78.7)	0.458	44 (74.6)	42 (71.2)	0.679
LVI	4 (6.1)	4 (2.2)	0.126	3 (5.1)	2 (3.4)	0.648
STAS	5 (7.6)	5 (2.7)	0.086	3 (5.1)	3 (5.1)	1.000
Ki‐67 index	10 (5.00, 27.50)	17.5 (5.00, 30.00)	0.217	8 (5, 15)	10 (5, 20)	0.821
Cell differentiation			0.018			0.582
Well	7 (10.6)	7 (3.8)		6 (10.2)	3 (5.1)	
Moderate	44 (66.7)	106 (57.9)		38 (64.4)	40 (67.8)	
Poor	15 (22.7)	70 (38.3)		15 (25.4)	16 (27.1)	

*Note*: Data are presented as No. (%) unless otherwise noted.

Abbreviations: CO, clinical observation; CEA, carcinoembryonic; CTR, consolidation tumor ratio; LLL, left lower lobe; LND, lymph node dissection; LUL, left upper lobe; LVI, lymphovascular invasion; PET‐CT, positron emission tomography‐computed tomography; pNx, unknown lymph node status; PSM, propensity score matching; RLL, right lower lobe; RML, right middle lobe; RUL, right upper lobe; STAS, spread through air spaces; TKI, tyrosine kinase inhibitor; VPI, visceral pleural invasion.

### 3.2 EGFR‐TKI distribution and safety

In the adjuvant TKI group after PSM, 38 (64.4%) patients chose to receive icotinib. Sixteen patients (27.1%) received gefitinib, and five (8.5%) were treated with osimertinib. Forty‐three patients (72.9%) were administered TKIs for 2 years, and 16 (27.1%) received adjuvant treatment for 1 year (Table 2). Treatment costs were $9200 per year for osimertinib, $5600 per year for icotinib, and $2000 per year for gefitinib. Adverse events of any grade were found in 26 (68.4%) patients who received icotinib, 14 (87.5%) patients who received gefitinib, and four (80%) patients who were administered osimertinib. Two patients (12.5%) switched from gefitinib to icotinib because of an unbearable rash during the first 2 months. None of the patients discontinued EGFR‐TKI therapy owing to the low occurrence rate of treatment‐related serious adverse events.

**TABLE 2:** Distribution of EGFR‐TKIs and adverse events after PSM.

Distribution	Icotinib (*n* = 38)	Gefitinib (*n* = 16)	Osimertinib (*n* = 5)
Duration			
1 year	8 (21.1)	5 (31.25)	3 (60)
2 years	30 (78.9)	11 (68.75)	2 (40)
3 years	0	0	0
Economy cost (USD/year)	5600	2000	9200
Adverse events			
Rash	14 (36.8)	8 (50)	2 (40)
Paronychia	7 (18.4)	4 (25)	2 (40)
Interstitial pneumonia	0	0	0
Platelet count decrease	0	0	0
Diarrhea	9 (24.7)	6 (37.5)	2 (40)
Nausea	2 (5.3)	1 (6.25)	1 (20)
Decreased appetite	1 (2.6)	0	1 (20)
Grade ≥3	2 (5.3)	3 (18.75)	0

*Note*: Data are presented as No. (%) unless otherwise noted.

Abbreviations: EGFR‐TKIs, epidermal growth factor receptor tyrosine kinase inhibitors; PSM, propensity score matching; USD, US dollar.

### 3.3 Survival outcomes

**FIGURE 2:**
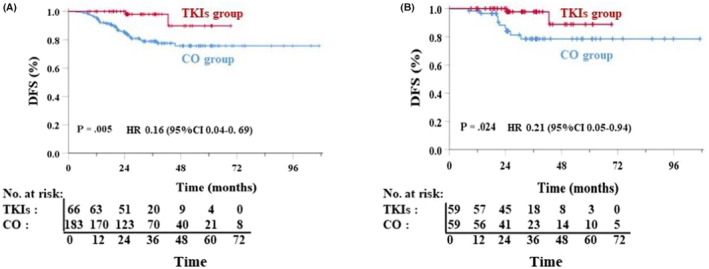
Kaplan–Meier plots of disease‐free survival in the entire cohort (A) and the matched cohort (B). DFS, disease‐free survival.

The median follow‐up time was 30.8 months (range: 7–107 months). In the entire cohort, 15 patients died, and 39 patients had experienced recurrence or metastasis by the last follow‐up. Two patients in the adjuvant TKI group experienced recurrence or metastasis. One patient received chemotherapy plus immunotherapy, and another patient received osimertinib in the presence of the *EGFR* p.Thr790Met resistance mutation. One patient in the TKI group died of a cardiovascular event. Thirty‐seven patients in the CO group experienced recurrence or metastasis. Twenty‐eight (75.7%) patients received first‐line EGFR‐TKIs, and nine (24.3%) patients received chemotherapy with or without immunotherapy. One patient died of myocardial infarction, one of laryngocarcinoma, and 12 of lung cancer‐related causes in the CO group. Among the matched 118 patients, 2 (3.4%) patients in the TKI group and 10 (16.9%) patients in the CO group experienced disease relapse. No brain metastases were observed in the TKI group, whereas 2 patients experienced central nervous system metastasis in the CO group. The 2‐year DFS rates were 98.3% in the TKI group and 86.4% in the CO group (HR: 0.12; 95% CI: 0.02–0.96; *p* = 0.032). The 3‐year DFS rates were 98.3% in the TKI group and 83.0% in the CO group (HR: 0.10; 95% CI: 0.01–0.78; *p* = 0.008). Differences between the two groups were found in the entire cohort (*p* = 0.005) (Figure 2A), as well as the matched cohorts (*p* = 0.024) (Figure 2B). The median DFS and OS data were immature owing to the short follow‐up period.

### 3.4 Analysis of DFS factors

Some variables, such as age, poor cell differentiation, micropapillary component, STAS, and targeted therapy, were significantly associated with DFS in the univariate analysis (*p* = 0.048, *p* = 0.009, *p* = 0.044, *p* = 0.012, and *p* = 0.041, respectively) (Table 3). Additional multivariate analysis showed that adjuvant EGFR‐TKIs was an independent factor for DFS (HR: 0.211; 95% CI: 0.045–0.979; *p* = 0.047), as well as poor cell differentiation (HR: 5.256; 95% CI: 1.648–16.769; *p* = 0.005), and STAS (HR: 5.612; 95% CI: 1.137–27.700; *p* = 0.034).

**TABLE 3:** Univariate and multivariate cox regression analysis of DFS factors.

DFS predictor	Univariate analysis		Multivariate analysis	
	HR (95% CI)	*p*	HR (95% CI)	*p*
Age	1.074 (1.001–1.153)	0.048	1.100 (0.974–1.243)	0.125
Sex	2.637 (0.835–8.331)	0.099	0.427 (0.069–2.634)	0.359
Smoking history	2.224 (0.600–8.243)	0.232	3.218 (0.423–24.477)	0.259
CEA abnormality	1.494 (0.400–5.572)	0.550	0.608 (0.091–4.074)	0.608
Tumor diameter	1.023 (0.944–1.108)	0.579	0.917 (0.794–1.060)	0.241
CTR	36.066 (0.164–7908.434)	0.192	16.924 (0.018–16,107.560)	0.419
Poor cell differentiation	4.636 (1.470–14.618)	0.009	5.256 (1.648–16.769)	0.005
Mucous component	1.573 (0.202–12.216)	0.665	0.476 (0.012–18.997)	0.693
Micropapillary component	3.253 (1.031–10.260)	0.044	3.960 (0.458–34.256)	0.211
Solid component	2.515 (0.550–11.497)	0.234	2.628 (0.072–95.530)	0.598
STAS	7.192 (1.533–33.739)	0.012	5.612 (1.137–27.700)	0.034
VPI	0.662 (0.199–2.202)	0.501	6.223 (0.532–72.805)	0.145
LVI	2.407 (0.311–18.653)	0.401	8.033 (0.325–198.680)	0.203
pNx	1.533 (0.336–7.005)	0.582	0.222 (0.021–2.395)	0.215
Targeted therapy	0.205 (0.045–0.939)	0.041	0.211 (0.045–0.979)	0.047

Abbreviations: CI, confidence interval; CEA, carcinoembryonic; CTR, consolidation tumor ratio; DFS, disease‐free survival; HR, hazard ratio; LVI, lymphovascular invasion; pNx, unknown lymph node status; STAS, spread through air spaces; VPI, visceral pleural invasion.

## 4 DISCUSSION

To the best of our knowledge, this real‐world study is the first to evaluate the efficacy and safety of adjuvant EGFR‐TKIs for stage IB lung adenocarcinoma based on the 8th edition AJCC/UICC stage classification, which demonstrated that adjuvant targeted therapy could significantly improve the 3‐year DFS rate (98.3% vs. 83.0%, respectively; HR: 0.10; 95% CI: 0.01–0.78; *p* = 0.008) compared with clinical observation, regardless of whether gefitinib, icotinib, or osimertinib were administered. None of the patients discontinued TKIs owing to the low occurrence rate of treatment‐related serious adverse events. However, the median DFS and OS data were immature owing to the relatively short follow‐up period.

Precision medicine plays a leading role in lung cancer healthcare. With the groundbreaking progress made in targeted therapy for metastatic NSCLC, many clinical trials have focused on early stage and locally advanced NSCLC.^19^, ^20^, ^24^, ^25^, ^26^ As described previously, the ADAURA trial revealed that adjuvant osimertinib administered for 3 years after chemotherapy could reduce the risk of relapse or death by 61% in the stage IB subgroup.^19^ The CORIN study showed that adjuvant icotinib for 1 year significantly improved the 3‐year DFS rate (95.3% vs. 86.7%, respectively; HR: 0.20; 95% CI: 0.04–0.89; *p* = 0.018).^20^ However, there were 50 patients in stage IB according to the latest edition of lung cancer stage classification in the ADAURA trial, comprising patients who received adjuvant chemotherapy and sequential osimertinib.^27^ Furthermore, the number of real stage IB patients in the CORIN study could not be assessed.

Nevertheless, based on the inspring findings of the ADAURA trial, the NCCN guidelines recommend that high‐risk stage IB patients receive reasonable adjuvant chemotherapy or osimertinib, which other guidelines do not follow due to the lack of accumulated evidence. High‐risk factors include poorly differentiated tumors, LVI, wedge resection, VPI, and pNx.^15^ In real‐world clinical practice, adjuvant therapy is not recommended by most medical and surgical oncologists. Previous studies have demonstrated that micropapillary and solid components correlate with early recurrence, multirecurrence, and poor survival.^28^, ^29^ Cellular heterogeneity is a fundamental feature of solid tumors, especially in locally advanced or metastatic NSCLC,^30^ which may elevate the recurrence rate and reduce the treatment response rate. We believe that intratumoral heterogeneity also exists in early stage NSCLC,^31^ which may be attributed to the micropapillary and solid components. STAS was recognized as a new pattern of invasion in lung adenocarcinoma by the WHO in 2015, showing poor prognosis.^32^ Numerous studies have revealed the poor prognostic significance of VPI, which was also shown by the International Association for the Study of Lung Cancer even after adjusting for tumor size.^21^, ^33^ Thus, most patients enrolled in our study had one or more high‐risk factors, including VPI, STAS, micropapillary components, and solid components. In the past few years, studies have found that MRD is associated with reduced DFS, even in patients undergoing radical resection.^5^, ^34^ However, we did not detect MRD using circulating tumor DNA, which was deemed an earlier prediction of relapse than traditional CT.^35^ However, this method is still experimental, with high costs and limited sensitivity in stage I NSCLC. As described in the results, two events occurred in the TKI group, and 10 events occurred in the observation group, even though the matched cohort in our study consisted of nearly half of the patients whose tumors were ≤3 cm upstaged to IB through VPI. A 90% reduction in the risk of recurrence was observed at 36 months; similar results were observed in the ADAURA and CORIN studies. Furthermore, we verified that adjuvant targeted therapy, poor cell differentiation, and STAS were independent risk factors for DFS. Therefore, the clinical significance of adjuvant EGFR‐TKIs in stage IB lung adenocarcinoma may be underestimated.

Previous randomized controlled trials (RCTs) have reported significantly longer DFS among patients who received adjuvant EGFR‐TKIs, compared with those who received chemotherapy. Two years of erlotinib treatment for stage IIIA NSCLC in the EVAN trial showed 81.4% 2‐year DFS, compared with 44.6% in the chemotherapy group.^25^ Patients with stage II‐IIIA NSCLC who received gefitinib for 24 months achieved 39.6% 3‐year DFS, compared with 32.5% in the chemotherapy group.^24^ The EVIDENCE trial showed that the 3‐year DFS in stage II‐IIIA patients was 63.9% with 2 years of icotinib treatment, compared with 32.5% with standard chemotherapy.^26^ TKIs were administered for 2 years in the EVAN, CTONG1104, and EVIDENCE trials, whereas 3 years was the standard in the ADAURA study.^19^ TKI resistance is an unavoidable issue for most patients, and most recurrence events occurred 24–36 months postoperatively. TKI treatment duration for locally advanced disease is still non‐uniform, and whether a longer adjuvant treatment time could postpone relapse and improve OS remains debatable. In our real‐world medical practice, 43 patients (72.9%) received TKIs for 2 years, while 27.1% (16/59) received targeted therapy for 1 year. None of the patients chose the 3‐year option. We believe that less than 2 years of TKIs administration is sufficient for patients with stage I NSCLC, although further evidence is needed to verify this. Frustratingly, the DFS advantage could not eventually translate into an OS benefit in the CTONG1104 trial.^24^ This may be attributed to subsequent therapies, including chemotherapy, radiotherapy, immunotherapy, palliative surgery, and EGFR‐TKI, which may greatly influence the final OS.^19^, ^24^, ^36^ In our study, 75.7% (28/37) of the patients who experienced postoperative recurrence in the CO group received subsequent EGFR‐TKI therapy, and the remaining patients received chemotherapy with or without other treatments. Unfortunately, 12 patients died of lung cancer‐related causes, which was not observed in the TKIs group. This indicated a trend, although it was not statistically significant. With longer follow‐up, we will be able to determine whether adjuvant targeted therapy can improve survival in stage IB lung adenocarcinoma, compared with CO. The ADAURA trial demonstrated that osimertinib can significantly reduce the risk of brain metastasis, compared with first‐generation TKIs.^19^ Our real‐world study failed to discover this result because of the relatively fewer patients treated with osimertinib, and with more recruited patients may confirm this conclusion.

Regarding the safety of EGFR‐TKIs, the occurrence rate of adverse effects was consistent with that in previous trials.^19^, ^24^, ^25^, ^26^ Rash and diarrhea were the most common adverse events, regardless of whether icotinib, gefitinib, or osimertinib was used. Adjuvant‐targeted therapy was safe and tolerable, although two patients who received icotinib and one patient who received gefitinib experienced grade 3 or worse adverse events. None of the patients discontinued EGFR‐TKIs owing to the low incidence of treatment‐related serious adverse events. Targeted therapies, including icotinib, gefitinib, and osimertinib, are not approved by the Chinese National Medical Products Administration for stage I NSCLC. Patients willing to take TKIs had to pay the fees themselves. Treatment costs per year amounted to $9200 for osimertinib, $5600 for icotinib, and $2000 for gefitinib. Patient economic burden should also be considered when recommending treatment.

Our study had several limitations. First, the non‐RCT nature resulted in a slightly heterogeneous study population, and the number of enrolled patients was relatively low, both of which could be overcome through further RCTs with a large number of patients. Second, the difference between first‐generation (icotinib and gefitinib) and third‐generation (osimertinib) TKIs may influence survival results. Additionally, the different oral durations of TKIs may also be a confounder. Third, our study had a relatively short follow‐up time, which may not have provided sufficient OS information.

## 5 CONCLUSIONS

Adjuvant EGFR‐TKIs could significantly improve DFS among patients with stage IB lung adenocarcinoma, compared with CO, with a safe and tolerable profile.

## AUTHOR CONTRIBUTIONS

**leilei shen:** Conceptualization (equal); data curation (equal); methodology (equal); writing – original draft (equal). **Juntang Guo:** Data curation (equal); writing – review and editing (equal). **Weidong Zhang:** Formal analysis (equal). **Lianbin Zhang:** Conceptualization (equal); supervision (equal); writing – review and editing (equal). **Xi Liu:** Conceptualization (equal); supervision (equal); writing – review and editing (equal). **Tao Wang:** Conceptualization (equal); supervision (equal); writing – review and editing (equal). **Tao Zhang:** Conceptualization (equal); supervision (equal); writing – review and editing (equal). **Chaoyang Liang:** Conceptualization (equal); supervision (equal); writing – review and editing (equal). **Yang Liu:** Conceptualization (equal); project administration (equal); supervision (equal); writing – review and editing (equal).

## FUNDING INFORMATION

This work was supported by the Special Science and Technology Projects for university and medical institutions of Sanya City (No. 2021GXYL44).

## CONFLICT OF INTEREST STATEMENT

The authors have no conflict of interest.

## ETHICS STATEMENT

The study was approved by the Ethics Committee of the Chinese People's Liberation Army General Hospital (S2021‐701‐01). All patients signed an informed consent form.

## Data Availability

The data that support the findings of this study are available on request from the corresponding author. The data are not publicly available due to privacy or ethical restrictions
